# Con-AAE: contrastive cycle adversarial autoencoders for single-cell multi-omics alignment and integration

**DOI:** 10.1093/bioinformatics/btad162

**Published:** 2023-03-28

**Authors:** Xuesong Wang, Zhihang Hu, Tingyang Yu, Yixuan Wang, Ruijie Wang, Yumeng Wei, Juan Shu, Jianzhu Ma, Yu Li

**Affiliations:** Department of Computer Science and Engineering, The Chinese University of Hong Kong (CUHK), Hong Kong SAR 999077, China; The Chinese University of Hong Kong (CUHK) Shenzhen Research Institute, Nanshan, Shenzhen 518057, China; School of Software Engineering, University of Science and Technology of China (USTC), Hefei 230026, China; Department of Computer Science and Engineering, The Chinese University of Hong Kong (CUHK), Hong Kong SAR 999077, China; Department of Mathematics, The Chinese University of Hong Kong (CUHK), Hong Kong SAR 999077, China; Department of Information Engineering, The Chinese University of Hong Kong (CUHK), Hong Kong SAR 999077, China; Department of Computer Science and Engineering, The Chinese University of Hong Kong (CUHK), Hong Kong SAR 999077, China; Department of Computer Science and Engineering, The Chinese University of Hong Kong (CUHK), Hong Kong SAR 999077, China; Department of Computer Science and Engineering, The Chinese University of Hong Kong (CUHK), Hong Kong SAR 999077, China; Department of Statistics, Purdue University, West Lafayette, IN 47907, United States; Department of Electrical Engineering, Tsinghua University, Beijing 100084, China; Institute for AI Industry Research, Tsinghua University, Beijing 100084, China; Department of Computer Science and Engineering, The Chinese University of Hong Kong (CUHK), Hong Kong SAR 999077, China; The Chinese University of Hong Kong (CUHK) Shenzhen Research Institute, Nanshan, Shenzhen 518057, China

## Abstract

**Motivation:**

We have entered the multi-omics era and can measure cells from different aspects. Hence, we can get a more comprehensive view by integrating or matching data from different spaces corresponding to the same object. However, it is particularly challenging in the single-cell multi-omics scenario because such data are very sparse with extremely high dimensions. Though some techniques can be used to measure scATAC-seq and scRNA-seq simultaneously, the data are usually highly noisy due to the limitations of the experimental environment.

**Results:**

To promote single-cell multi-omics research, we overcome the above challenges, proposing a novel framework, contrastive cycle adversarial autoencoders, which can align and integrate single-cell RNA-seq data and single-cell ATAC-seq data. Con-AAE can efficiently map the above data with high sparsity and noise from different spaces to a coordinated subspace, where alignment and integration tasks can be easier. We demonstrate its advantages on several datasets.

**Availability and implementation:**

Zenodo link: https://zenodo.org/badge/latestdoi/368779433. github: https://github.com/kakarotcq/Con-AAE.

## 1 Introduction

**Figure 1.**
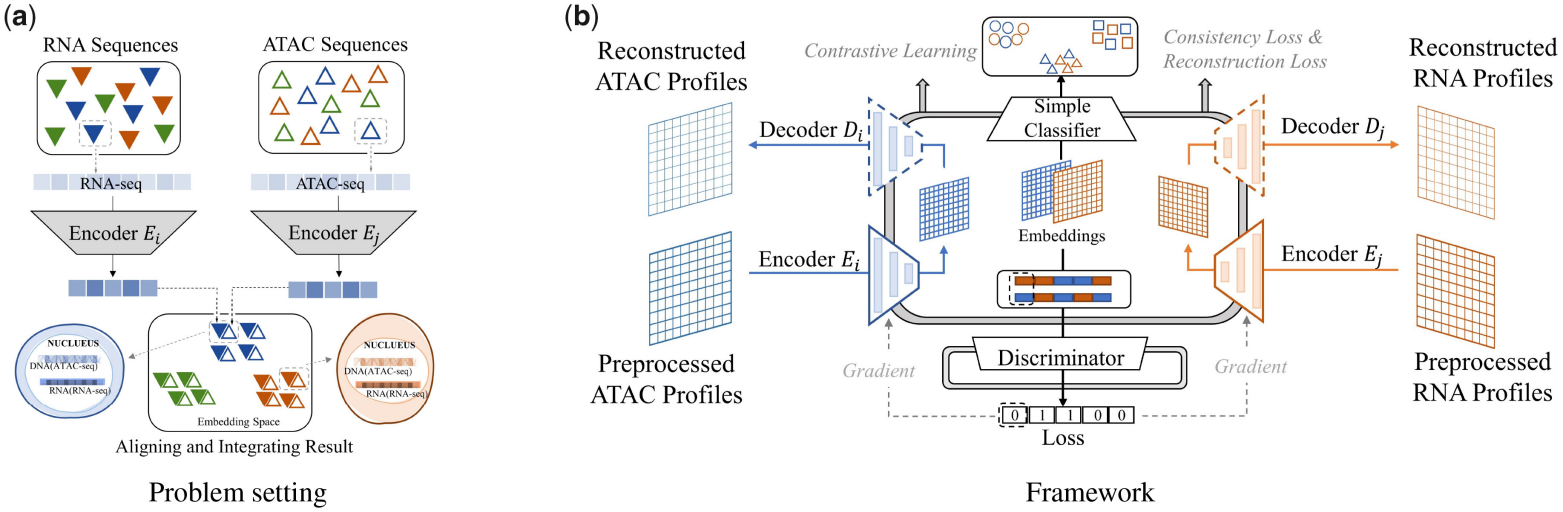
(a) scRNA-seq and scATAC-seq data measure different aspects of the same cell. We aim at identifying the correspondence between the two kinds of data from the same set of cells. (b) The Con-AAE framework uses two autoencoders to map the two kinds of sequence data into two low-dimensional manifolds, forcing the two spaces to be as unified as possible with the adversarial loss and latent cycle-consistency loss. We train the models without pairwise information for the alignment task but consider the data noise explicitly by utilizing self-supervised contrastive learning. We feed the annotated data for the integration task to help the model learn

Single-cell multi-omics methods promise great opportunities to understand the cellular system more comprehensively. To achieve that, we should integrate multi-omics data, which describe cells from different perspective. Profiling multi-omics for the same set of single cells have become available (Gala et al. 2021), such as single-cell RNA sequencing (scRNA-seq) and single-cell Assay for Transposase Accessible Chromatin sequencing (scATAC-seq), which describe the same cell from different perspectives. However, these techniques are not widely used due to the low sensitivity of one of the data modalities. Consequently, lots of computational methods have been proposed to integrate multi-omics data (Fig. 1a). We term this task as ‘integration’. More specifically, we want to obtain the high-throughput paired multi-omics data for every single cell, referred to ‘alignment’. On the other hand, even for data within the same modality, the data distribution can be inconsistent because of the subtle differences in measurement processes (Cao et al. 2018). This is the basic problem to be considered in the integration process.

Some computational methods have been proposed to deal with these two crucial but challenging problems, aligning and integrating data from different omics. People usually integrate and align multi-omics data in the learned low-dimensional embedding space using dimension reduction techniques, such as principal component analysis (PCA) (Bersanelli et al. 2016; Argelaguet et al. 2018) and non-linear successors of the classic canonical correlation analysis (CCA) (Stanley et al. 2020). The typical examples are Seurat (Stuart et al. 2019) and deep classic canonical correlation (DCCA) (Andrew et al. 2013). Seurat (Stuart et al. 2019) relies on the linear mapping of PCA and aligns the embedding vectors based on linear methods Mutual Nearest Neighbors and CCA, which weaken its ability to handle non-linear geometrical transformations across cellular modalities (Cao et al. 2022). DCCA can be effective for non-linear transformation benefiting from deep learning. However, according to the results of our experiments, it is not robust enough when the signal-to-noise ratio (SNR) is low. We also try Maximum Mean Discrepancy (MMD) (Borgwardt et al. 2006) to replace CCA in the embedding space, but the performance is also not good enough. Several methods requiring no correspondence information are derived under advanced machine-learning techniques, such as Pamona (Cao et al. 2022), MATCHER (Welch et al. 2017), MMD-MA (Singh et al. 2020), UnionCom (Cao et al. 2020), and SCOT (Demetci et al. 2022). Although these methods are unsupervised and achieve good performance with encouraging results (Demetci et al. 2022), they are not robust enough to noise. Deep learning methods are promising to provide alignment and transfer learning between datasets (Li et al. 2019, 2020). Deep generative models, such as CycleGAN (Zhu et al. 2017), MAGAN (Wang et al. 2017), RadialGAN (Yoon et al. 2018), and starGAN (Choi et al. 2018), are used to learn a non-linear mapping from one domain to another and achieve great performance on some single modality task. But the above transitions are almost within the same modality and can be disturbed by noise or sparsity in the data (Stanley et al. 2020). The scenario of multi-omics translation and alignment is much more complicated. Some other works propose models to align multi-omics data based on multiple autoencoders (Ma and Zhang 2019; Zhang et al. 2019; Dai Yang et al. 2021). However, such methods also can be seriously affected by noise or sparsity, which is a fundamental characteristic of single-cell data.

To promote the single-cell multi-omics data analysis, we propose a framework based on Contrastive cycle adversarial Autoencoders (Con-AAE), which can integrate multi-omics data (Fig. 1b).

Con-AAE uses two autoencoders to map two modality data into two low-dimensional manifolds under the constrain of adversarial loss, trying to develop representations for each modality that are separated but cannot be identified by an adversarial network in a coordinated subspace (Guo et al. 2019). However, only using the adversarial loss may lead to model collapse. To avoid the problem, we further propose a novel cycle-consistency loss. For instance, we have two autoencoders for two modalities, scATAC-seq data and scRNA-seq data. The embedding produced by the scRNA-seq encoder will go through the scATAC-seq decoder and encoder successively to produce another cycled embedding. We can check the consistency between the original embedding and the cycled embedding. In addition to the above two loss terms, we train the models without pairwise information for the alignment task but consider the data noise explicitly by taking advantage of self-supervised contrastive learning. For the integration task, we train the framework with annotated data. We extensively perform experiments on four real-world datasets, and a group of simulated datasets consisting of various distributions. The four real-world datasets consist of scATAC-seq and scRNA-seq data from the same set of cells. The comprehensive experiments on both simulated and real-world datasets show that our method has better performance and is more robust than the other state-of-art methods.

## 2 Materials and methods

In this section, we give our framework in details below with Fig. 1b, which illustrates the whole pipeline. To start with, we formalize the integration problem as,


(1) $$\forall R\in P_{R}\;\mathrm{and}\;\forall A\in P_{A},D(f(R))=D(h(A)).$$

We denote *R* as vectors of scRNA-seq domain and *A* as vectors of scATAC-seq domain. We would like to find two mappings *f* and *h* such that for any samples from *R* and *A*, *f* and *h* would maps the scRNA-seq profile and scATAC-seq profile to a coordinated subspace, where a discriminator D cannot distinguish *f*(*R*) and *h*(*A*). Due to the disparity between the original distributions of different omics data being complicated and non-linear, how to find a pair of *f* and *h* is a major problem.

In addition to map scRNA-seq data and scATAC-seq data to a coordinated subspace. The identity labels are available, and, therefore, it can be trained in a supervised way to maintain the cluster structure of cells. For ∀x∈{scRNA-seq embedding}∪{scATAC-seq embedding}, we want to train a classifier *g* such that,


(2) g(x)=label(x).

Our main model is built upon the framework of Adversarial Auto-Encoders (Makhzani et al. 2016) specialized for modality task, integrating our novel embedding consistency module. The intuition behind is that multi-omics from a single-cell data should obtain commonality, and their mappings could live in a coordinated subspace, which therefore makes alignment and integration possible.

### 2.1 Adversarial autoencoders

**Figure 2.**
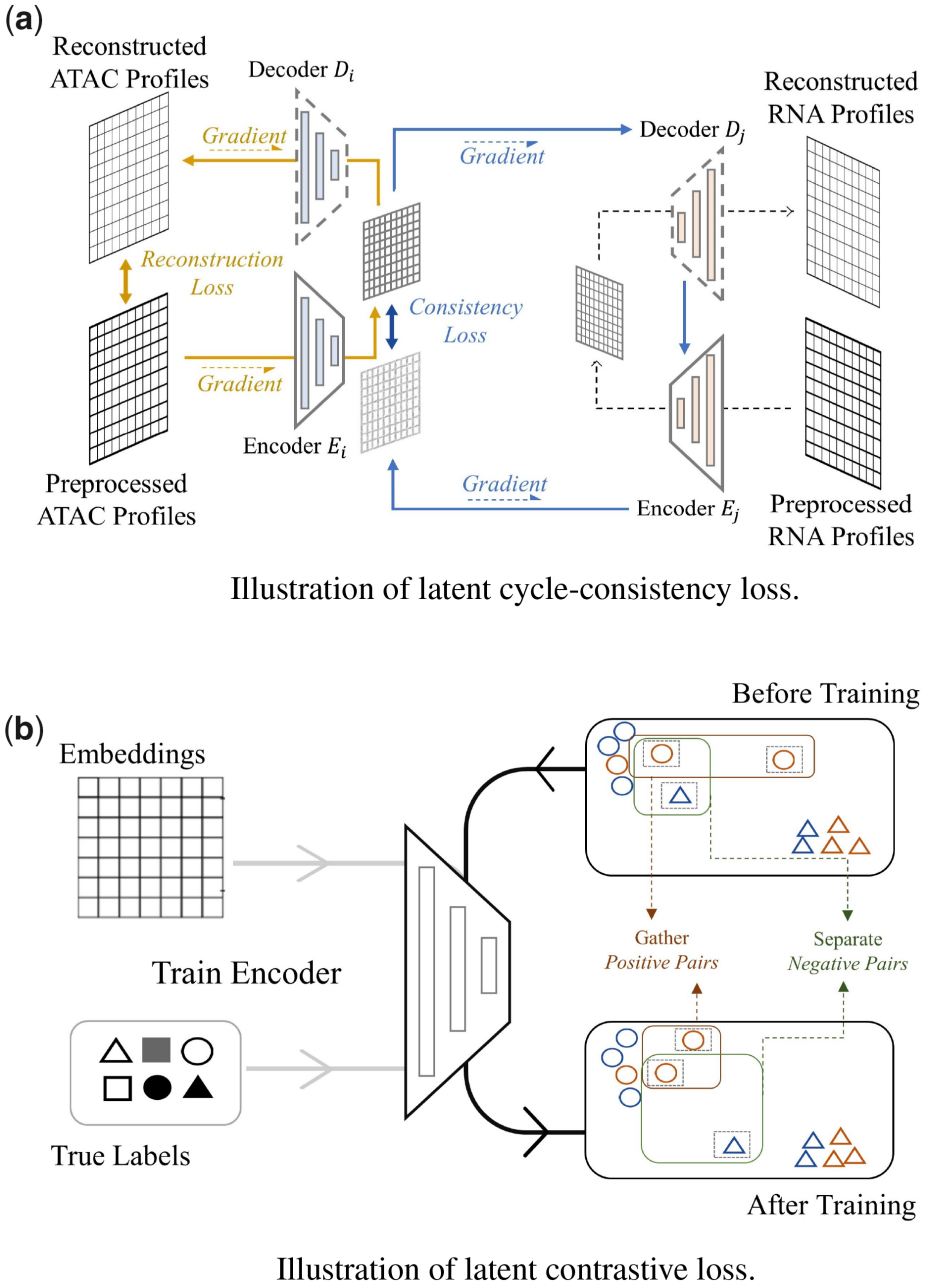
(a) The embedding produced by the first encoder will go through the second decoder and encoder successfully to produce another cycled embedding. We can check the consistency between the original embedding and the cycled embedding. (b) The contrastive loss minimizes the distance between positive pairs and maximizes the distance between negative pairs. This loss makes our method more robust to noise

The usage of adversarial autoencoders aims to constrain embeddings for each modality in a coordinated subspace, where embeddings mapped from different omics are close to each other. Therefore, as shown in Fig. 2b, we are using a coupled set of encoders $\left\{E_{i},E_{j}\right\}$ (Hira et al. 2021) to map {scATACs-eq, scRNA-seq} into manifolds $\left\{Z_{i},Z_{j}\right\}$, and decoders $\left\{D_{i},D_{j}\right\}$ could decode the embedded manifolds back to the original distribution. The reconstruction loss is defined as follows, whereas *d* stands for indicated distance in the embedding space. Discriminator D tries to align these embedded manifolds and works in the sense that input $x\in Z_{j},\;D(x)=1$ and $x\in Z_{i},\;D(x)=0$.


(3) $$\begin{array}{cc} L_{\mathrm{recon}} & =E_{x∼p_{\mathrm{rna}}}d(x,D_{j}(E_{j}(x)))\\ & +\;E_{x∼p_{\mathrm{atac}}}d(x,D_{i}(E_{i}(x))), \end{array}$$

(4) $$\begin{array}{cc} L_{\mathrm{adv}} & =E_{x∼p_{\mathrm{rna}}}[\text{log}\;D(E_{j}(x))]\\ & +\;E_{x∼p_{\mathrm{atac}}}[\text{log}(1-D(E_{i}(x)))]. \end{array}$$

The above losses *L*_recon_ and *L*_adv_ are trained together with the same weights.

### 2.2 Cycle-consistency loss

The backbone framework enforces the embedding manifolds to align gradually. However, a critical problem underlying is that since scRNA-seq and scATAC-seq data are sparse in a high dimensional domain, the training procedure above only aligns and trains on those regions where the data exist.

For instance, if a region *A* in the embedding space around $E_{j}(x^{\prime }),x^{\prime }\in${scRNA-seq} does not involve any existing $E_{i}(x),x\in${scATAC-seq}. Then, neither the decoder *D_i_* nor the encoder *E_i_* is trained on *A*, thus they would not compute in a “reverse” mapping way, and the result of $E_{i}D_{i}E_{j}(x)$ would be unreasonable or may not lie on the aligned manifold. This critical problem causes the difficulty of inferring from scRNA-seq profile to scATAC-seq profile directly.

Therefore, we introduce a cycle-consistency loss shown in Fig. 2a (Zhu et al. 2017; Hu and Wang 2019) to resolve this problem,


(5) $$\begin{array}{cc} L_{\mathrm{cyc}} & =E_{x∼p_{\mathrm{rna}}}[d(E_{j}(x),E_{i}D_{i}E_{j}(x))]\\ & +\;E_{x∼p_{\mathrm{atac}}}[d(E_{i}(x),E_{j}D_{j}E_{i}(x))]. \end{array}$$

*L*_cyc_ aims to train the set of encoder–decoder on the domain where different omics data may not exist, which enforces the smoothness and consistency in those regions. In this way, we could compare the embedding of $E_{j}(x),x\in${scATAC-seq} directly with the existing scRNA-seq embedding around it.

### 2.3 Contrastive loss

The above framework works in an unsupervised manner such that the embedded latent manifolds of multi-omics align properly.

We could further improve our work on both tasks by taking advantage of the ground-truth cell type labels. The cell type labels could refer to biological cell types or the labels of data batches collected from different times or platforms. Following the idea of contrastive learning (Schroff et al. 2015; Han et al. 2022), we employ a contrastive loss in embedding space. It enforces smaller In-Batch distance and larger Between-Batch distance. In-Batch refers to different modality data collected from the same cluster and *vice versa*. We equally treat both modalities in contrastive training, which benefits the alignment task in the sense that multi-omics of the same single-cell data should obviously belong to the same cluster. We show that lowering the In-Batch distance indeed improves the alignment accuracy in the below ablation studies. On the other hand, contrastive training benefits integration by enabling the decision boundary to be smoother and more robust.

In practice, we first encode data from two modalities to the embedding space. Define the embedding by z∈Z. Given *z^a^* as anchor vector in embedding space, we select *z^p^* such that $\mathrm{argma}x_{z^{p}}\{d(z^{a},z^{p})\},\mathrm{label}\{z^{a}\}=\mathrm{label}\{z^{p}\}$, which is named hard positive. The intuition of hard positive is to find a vector furthest from the anchor within same cluster. Similarly, we have *z^n^* as hard negative such that $\mathrm{argmi}n_{z^{n}}\{d(z^{a},z^{n})\},\mathrm{label}\{z^{a}\}\neq \mathrm{label}\{z^{n}\}$. *z^n^* is defined as the closest vector from a different cluster. The objective immediately follows,


(6) $$d(z^{a},z^{p})+\alpha <d(z^{a},z^{n}),\forall (z^{a},z^{p},z^{n})\in Z.$$

Above, *α* is the margin defined accordingly by us. Thus, by the contrastive loss, we tend to optimize,


(7) $$\begin{array}{cc} L_{\mathrm{con}} & =E_{z^{a}∼Z}[d(z^{a},z^{p})-d(z^{a},z^{n})+\alpha ].\\ L_{\mathrm{con}} & =E_{x∼\{\mathrm{RNA}\}}[d(E_{j}(x),z^{p})-d(E_{j}(x),z^{n})+\alpha ]\\ & +\;E_{x∼\{\mathrm{ATAC}\}}[d(E_{i}(x),z^{p})-d(E_{i}(x),z^{n})+\alpha ]. \end{array}$$

Figure 2b shows that after training, instances within the same cluster are pushed towards each other, and those from the different clusters are forced away. Thus, the decision boundary of the labels tends to be smoother and more robust, which also benefits the alignment task.

### 2.4 Simple classifier

We introduce a simple classifier in the coordinated subspace to further promote the framework’s performance. This simple classifier takes both embeddings encoded from scRNA-seq and scATAC-seq as input and predicts their labels for cell types. As Fig. 1b shows, our model forces the embeddings from the same cell type into a cluster, making it easier to match embeddings from different modalities. We employ cross-entropy loss to optimize the simple classifier at the same time as we optimize the coupled autoencoders. We adapt the classifier as *C*. The predicted result for an embedding *z_k_* is a vector of 1×m shape. *m* is the number of cell types. Then, we have $C(z_{k})=[c_{1},\ldots ,c_{m}]$ and denote *c_k_* as the predicted probability of real cell types for *z_k_*. We try to minimize the loss *L*_classifier_ as: $\left\{Z_{i},Z_{j}\right\}$ is denoted as embeddings encoded from scRNA-seq and scATAC-seq.


(8) $$L_{\mathrm{classifier}}=E_{z_{k}∼\{Z_{i},Z_{j}\}}[-\ln c_{k}].$$

### 2.5 Training procedure

In the above sections, we proposed several losses related to different objectives. Following the training procedure of Generative Adversarial Nets (Dai Yang et al. 2021), we adopt a two-stage training scheme where *L*_adv_ and $L_{\mathrm{recon}},L_{\mathrm{cyc}},L_{\mathrm{con}},L_{\mathrm{classifier}}$ are trained separately as the pseudo-code in Algorithm 1.

Algorithm 1: Training Procedure

 **while** numbers of training iterations **do**

  **while** *k*_1_ steps **do**

   sample mini-batch $\left\{x_{1},x_{2},\ldots ,x_{m}\right\}$ from {scRNA-seq}

   sample mini-batch $\left\{y_{1},y_{2},\ldots ,y_{m}\right\}$ from {scATAC-seq}

  Search positives and negatives *z^a^*, *z^p^* for each $x_{1},\ldots ,y_{m}$.

   Update $E_{i},E_{j},D_{i},D_{j}$ by descending its stochastic gradient $\frac{1}{m}\nabla \left(L_{\mathrm{recon}}+L_{\mathrm{cyc}}+L_{\mathrm{con}}+L_{\mathrm{classifier}}-L_{\mathrm{adv}}\right)$

  **end while**

  **while** *k*_2_ steps **do**

   sample mini-batch $\left\{x_{1},x_{2},\ldots ,x_{m}\right\}$ from {scRNA-seq}

   sample mini-batch $\left\{y_{1},y_{2},\ldots ,y_{m}\right\}$ from {scATAC-seq}

   Update Discriminator D by descending its stochastic gradient $\frac{1}{2m}\nabla L_{\mathrm{adv}}$

  **end while**

 **end while**

In this way, the Discriminator D competes against the encoder–decoder $E_{i},E_{j},D_{i},D_{j}$ until the training ends and reaches the equilibrium.

## 3 Results

### 3.1 Evaluation criteria

We utilize two existing manners (Dai Yang et al. 2021) to evaluate integration and alignment, respectively. (i) We match samples from RNA-seq to scATAC-seq in the coordinated subspace. For each embedding encoded from scATAC-seq, we calculate the Euclidean distance between it and each embedding encoded from scRNA-seq in coordinated space and find the closest pair. We regard it as a correct match if they are from the same cell type. Then, we evaluate the integration performance by the fraction of such correct matching in the test set. (ii) Like step in (i), we still calculate the distance between samples from scRNA-seq and samples from scATAC-seq in the coordinated subspace. For each embedding encoded from scATAC-seq, instead of picking the nearest encoded scRNA-seq sample, we choose the *k* closest scRNA-seq embeddings. We calculate *k*-nearest neighbours accuracy as: where *n* is the cell numbers in test set. *R* is the set of RNA-seq samples and *A* is denoted as the set of ATAC-seq samples. We denote *r_i_* as a sample from *R*, *a_i_* as a sample from A. ${a^{\prime }}_{i}$ and ${r^{\prime }}_{i}$ are the encoded versions of *a_i_* and *r_i_*, respectively. $a_{i}^{k}$ contains the *k* nearest $r^{\prime }$ to ${a^{\prime }}_{i}$. We denote *k* as 10, 20, 30, 40, and 50, evaluating alignment performance. We call this evaluation way recall@k.


(9) $$\mathrm{kNN}(A,R)=\frac{\sum_{i}1\left({a^{\prime }}_{i}\in r_{i}^{k}\right)}{n},$$

### 3.2 Compared with SOTA

Instead of assuming all datasets share the same underlying structure or specifying parts of hyperparameters like some traditional machine-learning methods (Welch et al. 2017; Stuart et al. 2019; Cao et al. 2020; Demetci et al. 2022; Singh et al. 2020; Cao et al. 2022), we obtain more information from datasets with partial correspondence information (batch label or cell types label). We select several state-of-art methods based on deep learning like ours, including Cross-Modal (Dai Yang et al. 2021), Cross-Modal-anchor (pairwise information added), DCCA (Andrew et al. 2013), CycleGAN (Zhu et al. 2017), and scJoint (Lin et al. 2022). Moreover, we also compare our method with machine-learning methods for integration, including MOFA+ (Argelaguet et al. 2020), Seurat (Stuart et al. 2019), Pamona (Cao et al. 2022), MMD-MA (Singh et al. 2020), UnionCom (Cao et al. 2020), and SCOT (Demetci et al. 2022). For Seurat (Stuart et al. 2019), we convert scATAC-seq data to predicted gene count matrix by Cicero (Pliner et al. 2018) then integrate them. We apply Con-AAE and these methods on simulated and real-world datasets.

**Figure 3.**
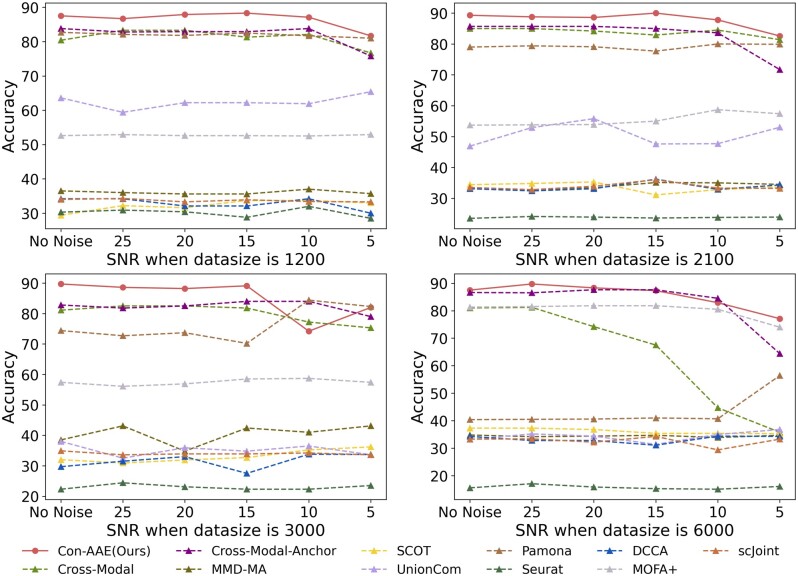
The figure shows the integration performance on 24 simulated datasets with various data sizes and SNR. The horizontal axis represents the SNR, and the vertical axis represents the percentage of correct integration. The Con-AAE outperforms other methods in most cases. As the SNR ratio decreases and the size of the dataset grows, the performance of all the methods degrades significantly. However, Con-AAE still has excellent performance, demonstrating its scalability and robustness

**Figure 4.**
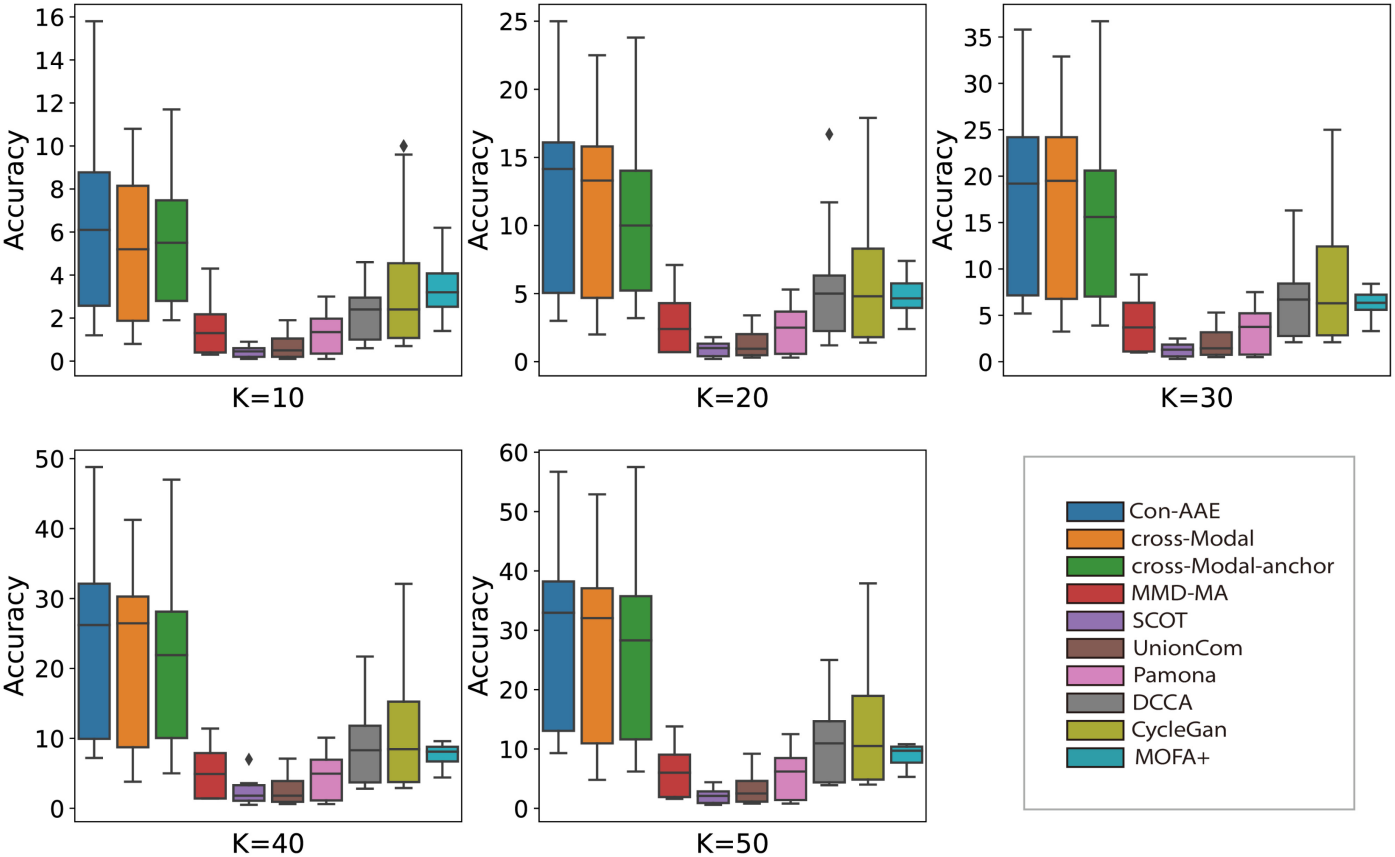
The box plot shows the alignment performance on 24 simulated datasets with various data sizes and SNR. In most cases, Con-AAE has almost the highest upper edge, lower edge, median, and upper and lower quartiles, which indicates that the overall performance distribution of Con-AAE is higher than that of other methods

We generate 24 sets of different sizes and SNRs. The generating process can be found in Supplementary Section B. The simulated datasets contain data of four different sizes, 1200, 2100, 3000, and 6000, each of which has six versions of SNR, 0, 5, 10, 15, 20, and 25, respectively. We implement all methods on the simulated datasets. The results in Fig. 3 show that Con-AAE’s performance is stable and has the best performance in most cases regardless of data size and SNR, demonstrating our method’s robustness and scalability. We also calculate the alignment performance on the simulated datasets. We set *k*=10, 20, 30, 40, and 50, so there are 120(5 × 24) results for each method. Figure 4 shows the performance of all results in box plots. We can see that the upper edge, lower edge, median, and upper and lower quartiles of Con-AAE are higher in most cases than other tools. Our method’s performance is almost consistent against different data sizes and noise levels. In contrast, the other methods may perform well in some settings but poorly in others. The results indicate that Con-AAE is robust and stable enough to have the potential to handle the complicated single-cell multi-omics alignment and integration problems with a low SNR ratio.

**Figure 5.**
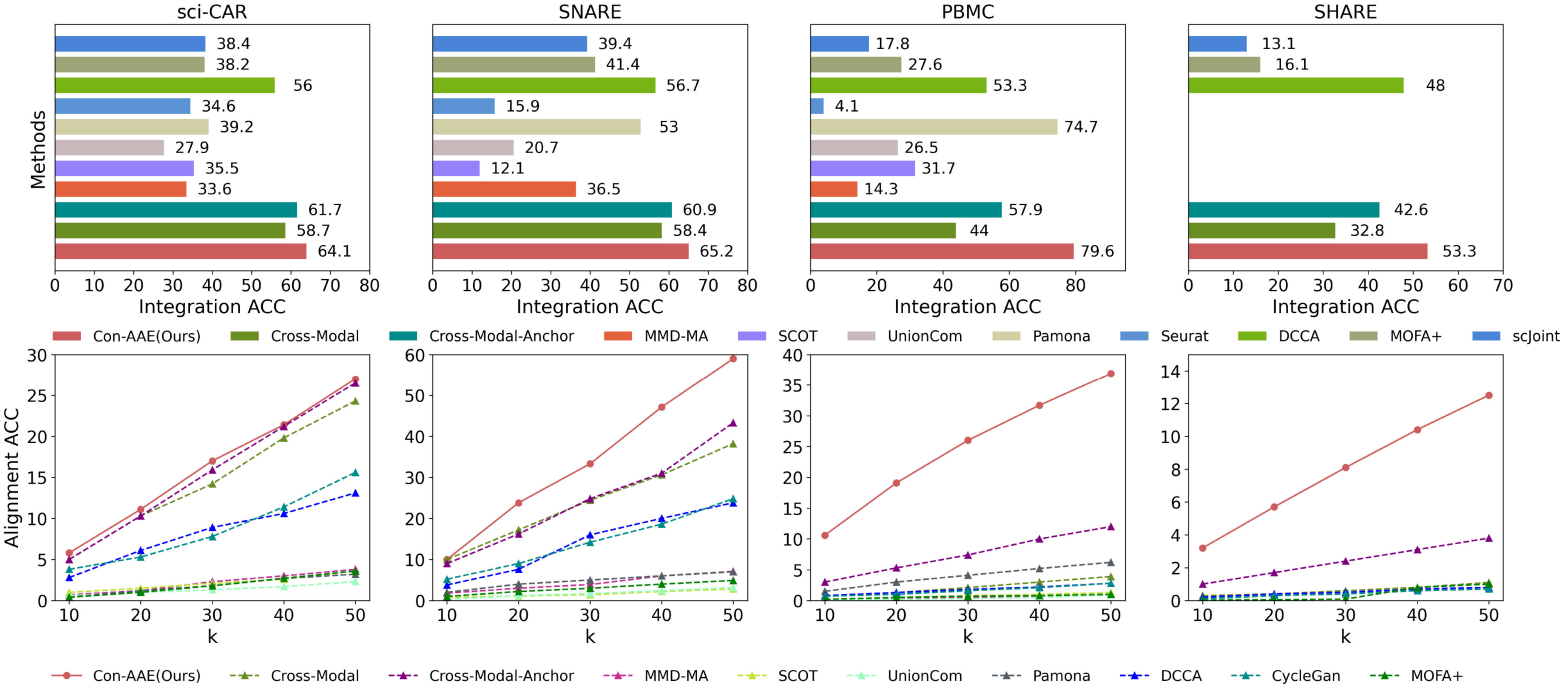
Con-AAE compares with SOTA methods on the four real-world datasets. The upside is the integration performance, and the downside is alignment performance. The horizontal axis of the upside and the vertical axis of the downside are percentages. Con-AAE has the best performance on both criteria. Note that the identification of cell pairwise correspondences between single cells is termed “anchor” (Stuart et al. 2019). Cross-Modal-anchor indicates that “anchor” information is provided when training Cross-Modal

We care about the methods’ performance on the real-world datasets the most, although the real-world datasets with ground-truth information are limited. Still, Con-AAE shows superior performance. On the sci-CAR dataset, Con-AAE outperforms the other methods by up to 36.2% on the integration task, as shown in the upper part of Fig. 5. For alignment, Con-AAE always has better performance than all the other methods no matter what *k* is (the bottom part of Fig. 5). On the SNARE-seq dataset, more obviously, Con-AAE also has dominant performance on each evaluation metric. The improvement on the integration task is up to 53.1% (Fig. 5). On the other hand, the performance on recall@k is better than others no matter what *k* is (Fig. 5). We also conduct all methods on more complex cases, more than 9000 cells of 19 cell types from 10X PBMC, which can be downloaded at https://support.10xgenomics.com/single-cell-multiome-atac-gex/datasets/1.0.0/pbmc_granulocyte_sorted_10k, and 34 774 cells of 23 cell types from SHARE-seq (Ma et al. 2020). Our results in Fig. 5 indicate that Con-AAE still performs excellently in more complex situations while most other tools get poorer performance. Due to insufficient memory processing technology, several tools cannot deal with large datasets like SHARE-seq. Again, Con-AAE is consistently better than the other competing methods.

### 3.3 Ablation studies

We perform comprehensive ablation studies on the sci-CAR dataset, and the results show the effectiveness of different components.

There are three parts in (Supplementary Table S6). The first part indicates there is no adversarial loss in embedding space. The second part indicates an MMD loss (Bińkowski et al. 2018) instead of an adversarial loss. And the last part indicates whether there is an adversarial loss in the embedding space. Most items in the third part are better than the corresponding items in the other two parts, demonstrating that the adversarial loss works better than MMD loss on this problem.

Five items represent different combination of loss functions in each part of (Supplementary Table S6). The first row represents the basic framework, consisting of two coupled autoencoders and a simple one-layer classifier. The anchor one means pairwise information provided, which indicates that it is a supervised learning model instead of an unsupervised one. “cyc” and “contra” denote cycle-consistency loss and contrastive loss, respectively. As shown in the table, adding “cyc” and “contra” improves the model. Apparently, Con-AAE has the best performance. Cycle-consistency loss and contrastive loss alone can improve the performance to some degree, but Con-AEE is more robust and has better scalability.

Impressively, Con-AAE has better performance even compared to some supervised methods with the pairwise information provided. Within Supplementary Table S6, we compare our approach with methods fed pairwise information. We train them using the pairwise information as the supervision for such methods. For Con-AAE, we still perform unsupervised learning using cycle-consistency loss and contrastive loss. Even without the supervised information, Con-AAE can still outperform the basic supervised anchor methods consistently on both tasks. It suggests that cycle-consistency loss and contrastive loss can force our model to learn a coordinated subspace for the two kinds of single-cell omics data, making the alignment and integration much easier. We also try to combine Con-AAE with the pairwise information. The supervised information can help our method further, but the degree is very slight (Supplementary Table S9). We suppose that in the real data, the pairwise information may contain noise, which is common in the single-cell field. Because of the contrastive loss, which makes Con-AAE a robust method, such weak supervision does not help our model too much.

### 3.4 Visualization

To further demonstrate our model’s performance and make integration the effect more intuitive, we transfer the label from scRNA-seq to scATAC when integrating them. Firstly, we encode scRNA-seq and scATAC-seq into a low-dimensional coordinated subspace with Con-AAE. Assuming the labels of scRNA-seq are known, for each embedding encoded from scATAC-seq, we assign it with the label by the nearest (calculated with Euclidean) scRNA-seq embedding. We conduct the transferring process on four real-world datasets test set with the help of Con-AAE and visualize embeddings by t-SNE (Van der Maaten and Hinton 2008). We can see that the transferred labels are almost consistent with real labels in Supplementary Fig. S1, which visually demonstrates the good integration by Con-AAE. In addition, the excellent clustering effect also reflects the power of contrastive learning. We also conduct the same process with other tools, as Supplementary Figs S2–S10 show. Most tools perform poorly on large datasets with more than 10 or even 20 cell types, while Con-AAE still performs well on complex datasets benefitting from the power of contrastive learning.

## 4 Discussion

In this article, we propose a novel framework, Con-AAE, aiming at integrating and aligning the multi-omics data at the single-cell level. On the one hand, our proposed method can map different modalities into a coordinated subspace with the help of an adversarial loss and a novel cycle-consistency loss. On the other hand, we apply a novel self-supervised contrastive loss in the embedding space to improve the robustness and scalability of the entire framework. Comprehensive experimental results on the simulated and real datasets show that the proposed framework can outperform the other state-of-the-art methods for both alignment and integration tasks. Detailed ablation studies also dissect and demonstrate the effectiveness of each component in the framework. Our method will be helpful for both the single-cell multi-omics research and the general multi-modality learning tasks in computational biology.

For future work, we aim to extend our work from a two-domain task to a multiple-domain study, allowing it to integrate and align multiple omics. Besides integration and alignment between sequence modalities, we intend to perform our method on different kinds of biological data, including but not limited to images, geometrical spatial structure, etc. Obviously, it is exciting to investigate the spatial transcriptomics data. We will also develop methods for translating modalities. By doing so, we hope to build a system that could benefit various downstream analyses in single-cell multi-omics and spatial multi-omics.

## Supplementary Material

btad162_Supplementary_Data

## Data Availability

All processed data except SHARE-seq can be found at: https://github.com/kakarotcq/Con-AAE/tree/main/data. SHARE-seq data can be found through GSE140203 on NCBI.
