# Supplementary material for: Con-AAE: contrastive cycle adversarial autoencoders for single-cell multi-omics alignment and integration
Source: Bioinformatics. 2023 Mar 28;39(4):btad162. doi: 10.1093/bioinformatics/btad162 (PMC10101696; doi:10.1093/bioinformatics/btad162)
Supplement: btad162_Supplementary_Data [file btad162_supplementary_data.pdf]

Contrastive Cycle Adversarial Autoencoders for Single-cell Multi-omics Alignment and Integration

XUESONG WANG [1,4,7], ZHIHANG HU [1], TINGYANG YU [2,3], YIXUAN WANG [1], RUIJIE WANG [1], YUMENG WEI [1], JUAN SHU [5], JIANZHU MA [6] AND YU LI [1, 4,*]

[1]Department of Computer Science and Engineering, The Chinese University of Hong Kong (CUHK), Hong Kong SAR, China,
[2]Department of Mathematics, The Chinese University of Hong Kong (CUHK), Hong Kong SAR, China,
[3]Department of Information Engineering, The Chinese University of Hong Kong (CUHK), Hong Kong SAR, China,
[4]The Chinese University of Hong Kong (CUHK) Shenzhen Research Institute, Hi-Tech Park, Nanshan, Shenzhen 518057, China,
[5]Department of Statistics, Purdue University, West Lafayette, IN 47907, United States,
[6]Department of Electrical Engineering and Institute for AI Industry Research, Tsinghua University, Beijing 100190, China,
[7]School of Software Engineering, University of Science and Technology of China (USTC), Hefei 230052, China.

This supplementary material provides more experimental results.

1. SUPPLEMENTARY MATERIALS

A. Real-world Dataset

We use two sets of single-cell multi-omics data generated by co-assays. The first dataset is generated using the sci-CAR assay [1]. For the single-cell ATAC-seq data, we download the processed data from [2], which are computed as described in [1]. Then we have a matrix of 1791×815. For the single-cell RNA-seq data, we pick the genes with $q - value > 0.05$ from the genes being differentially expressed [1], which forms a matrix of 1791×2613 genes. Such paired data are collected from human lung adenocarcinoma-derived A549 cells corresponding to 0-, 1-, or 3-hour treatment with Dexamethasone (DEX).

We denote the results of SNARE-seq [3] assay as the second dataset, which also consists of chromatin accessibility and gene expression. The data are collected from a mixture of human cell lines: BJ, H1, K562, and GM12878. We reduce the dimension of the data by PCA. The resulting matrix for scATAC-seq is of size 1047×1000 and 1047×500 for gene matrix.

We collect PBMC dataset from 10X genomics website, which contains 11909 cells with 15494 linked genes and 85468 linked peaks. We get the filtered version [4] containing 9631 cells, then we conduct PCA on it and get 2000 features for each omic.

From NCBI GSE140203, we get mouse skin datasets produced by SHARE-seq technique [5], which contains 34774 cells of 23 cell types. The raw scATAC data has 344592 peaks, and the raw scRNA data contains 23296 genes. We reduce both of them to 2000 dimensions by PCA.

B. Simulated Datasets

We simulate several datasets of different sizes inspired by the idea from splatter [6], which contain 1200, 2100, 3000, and 6000 cells. For first modality, we utilize three Gaussian distributions with different parameters to generate three batches, and the feature dimension is 1000. For second modality, we train a four-layer autoencoder with the first modality data and map them to 500 dimensions. After that, we randomly set around 40% of features to 0 for first modality data since the real-world multi-omics data matrix is usually very sparse. Considering the inevitable mismatch in the experiment, we randomly set around 10% mismatches in the datasets and shuffle all the pairs. Furthermore, we add noise to them with the SNR equal to 5, 10, 15, 20, and 25, along with the version without noise. Then, we have 24 simulated datasets here. Surely, the real-world multi-omics are more complicated than the simulated data, but the experimental results show that our method is sufficient to distinguish the performance of different methods.

We split all the datasets into training sets and test sets, with the first 80% as the training sets and the last 20% as test sets. Note that we shuffled the data before splitting them.

C. Neural Network Architectures

Usually, for the input layer, the number of neurons is equal to the feature dimension, and the number of neurons in the output layer is equal to the number of classification categories. Regarding the hidden layers, we usually use one or no hidden layer in easy tasks, such as simple classification. But for more complex scenes, we need more hidden layers. Referring to what Andrew Ng mentioned in the coursera course if multiple hidden layers are used, the number of neurons in each hidden layer should be the same. More hidden layers mean a more complex network, which is likely to improve the model's performance. Because more neurons can learn more complex information, we utilize such many hidden layers in autoencoder because scRNA-seq and scATAC-seq are pretty complex data containing a large amount of information. However, training the model with multiple hidden layers will take longer. So we

only design a few hidden layers for the discriminator network and one layer for simple classifier, it is not a difficult task to classify embedding from different omics in the coordinated subspace, and it is just used as a constraint condition to form a coordinated representative subspace. According to our empirical impressions and sensitivity above, we design our model like Table. S1, S2, S3.

Table S1. The architecture of Autoencoder. 'd' refers to the dimension of the input data. 50 is the dimension of the embedding space.

	Encoder	Decoder
Input Layer Size	d	50
Activation Function	LeakyReLU	LeakyReLU
Hidden Layer Size	d	100
Activation Function	LeakyReLU	LeakyReLU
Hidden Layer Size	d	d
Activation Function	LeakyReLU	LeakyReLU
Hidden Layer Size	100	d
Activation Function	LeakyReLU	LeakyReLU
Hidden Layer Size	50	d

Table S2. The architecture of discriminator network. The output of the discriminator is a number ranging from [0,1], representing the probability that the input embedding comes from a certain modality, then one minus output representing the possibility for the other one.

	Discriminator Network
Input Layer Size	50
Activation Function	LeakyReLU
Hidden Layer Size	100
Activation Function	LeakyReLU
Hidden Layer Size	1
Activation Function	Sigmoid

Table S3. The architecture of simple classifier network. The output size is equal to the number of cell types, each element of which represents the predicted possibility of corresponding cell types.

	Simple Classifier
Input Layer Size	50
Output Layer Size	n

D. Sensitivity analysis

With the help of Salib [7] (a python package for sensitivity analysis), we conducted a sensitivity analysis on six important hyperparameters.

We denote α as the margin in the contrastive learning, w as the weight of adversarial loss, contrastive loss, and consistency loss (these losses sharing the same weight), β as the weight of simple classifier loss. The parameters of the optimizer have a great influence on the overall model so we also pay attention to them. We utilize β_1 and β_2 representing coefficients for computing gradients and running averages of squared gradients in adam optimizer, lr as learning rate. $S1$ is the first-order sensitivity, which measures the contribution of a single parameter to the variance of the model outcome. $S2$ is the second-order sensitivity, measuring the

Table S4. First-order sensitivity of each hyperparmeter.

	range	$S1$	$S1_{conf}$	St	St_{conf}
α	[0.1,1]	-2.340087	1.566277	1.983067	1.145647
w	[1,10]	-0.013034	1.094778	0.318858	0.332082
β	[1,10]	0.220378	0.601713	0.259467	0.548236
β_1	[0.1,1]	0.579564	1.165850	1.908934	3.554098
β_2	[0.1,1]	-0.453080	1.404616	1.096837	1.687147
lr	[0.0001,0.1]	-1.013364	3.091715	1.862601	1.143023

Table S5. Second-order sensitivity of each hyperparmeter.

	$S2$	$S2_{conf}$
(α,w)	3.362682	0.742656
(α,β)	1.751124	2.725716
(α,β_1)	1.319802	3.148916
(α,β_2)	1.385512	2.511941
(α,lr)	2.842063	1.977569
(w,β)	-0.356346	1.211126
(w,β_1)	-1.138115	2.626966
(w,β_2)	0.083400	2.066652
(w,lr)	1.710122	1.959094
(β,β_1)	-0.368368	0.395337
(β,β_2)	-0.121538	0.406597
(β,lr)	0.366226	1.002690
(β_1,β_2)	-1.871891	3.086358
(β_1,lr)	0.689079	1.061860
(β_2,lr)	2.947162	3.178585

contribution of the interaction of two parameters to the variance of the output. St is total-order sensitivity, reflecting the contribution of the single parameter interacting with all other parameters to output variance. $S1_{conf}$, $S2_{conf}$, St_{conf} means confidence score respectively. Larger sensitivity value indicates greater impact on model performance. Table. S4 shows that β and β_1 are impactful to model, because their $S1$ scores are high, so we can choose these two parameters first. For other parameters, though their $S1$ scores are low, their St scores shows their impacts when incorporating with other parameters. We can see that $S2$ score in Table. S5 is high when α incorporating other five parameters, especially with w. We can adjust these two parameters at the same time. The $S2$ score of β_2 and lr is also pretty high, then we can tuning the model by changing them simultaneously.

E. Alblation study on simulated dataset

Table S6 and Table S7 display the ablation study on simulated data.

Table S6. Ablation Study of different components in Con-AAE and Comparison with other methods. Basic refers to Coupled AEs plus Simple classifier; adv refers to adversarial loss; mmd refers to mmd loss; anchor refers to pairwise information added; cyc refers to cycle-consistency loss; contra refers to contrastive loss.

Method	Integration ACC	Recall@k k=10	Recall@k k=20	Recall@k k=30	Recall@k k=40	Recall@k k=50
Basic	56.1	3.9	9.2	13.4	18.1	20.6
Basic_anchor	60.8	5.3	12	15.9	22.3	28.4
Basic_cyc	56.7	6.7	11.7	15.6	19.8	24.5
Basic_contra	58.9	**6.9**	12.5	15.6	21.7	27.3
Basic_contra_cyc	62.2	7.8	10.8	15.9	21.2	25.9
Basic_mmd	57.5	4.7	9.4	12.5	17.3	21.2
Basic_mmd_anchor	56.4	5.3	11.4	16.7	21.2	24.3
Basic_mmd_cyc	60.8	3.9	10	16.2	21.2	25.1
Basic_mmd_contra	57.5	5.8	**12.8**	15.6	24	27
Basic_mmd_contra_cyc	57.8	4.5	9.5	13.7	19.8	24.8
Basic_adv	58.3	4.4	10.3	14.2	17.5	23.1
Basic_adv_anchor	61.7	5	10.3	15.9	21.2	26.5
Basic_adv_cyc	58.7	5	10.3	14.2	19.8	24.3
Basic_adv_contra	60.61	4.4	9.4	13.6	18.4	25.4
**Con-AAE**	**64.1**	5.3	12	**17**	**22.6**	**28.4**

Table S7. Ablation study on the simulated datasets with various SNRs: basic plus adversarial loss (denoted as Basic_adv), basic plus adversarial loss and cycle-consistency loss method (denoted as Basic_adv_cyc), basic plus adversarial loss and contrastive loss (denoted as Basic_adv_contra) and basic plus adversarial loss, cycle-consistency loss, and contrastive loss (Con-AAE). Con-AAE's performance is very stable across different data sizes and SNRs, compared to the other baseline methods. SD is the abbreviation for standard deviation and AVG is the abbreviation for average value.

#Sample	Method	No Noise	SNR25	SNR20	SNR15	SNR10	SNR5	SD	AVG
1200	Basic_adv	80.4	83.3	83.3	81.3	82.1	76.7	2.47	81.18
	Basic_adv_cyc	83.3	83.3	83.8	83.3	84.2	82.3	**0.64**	83.36
	Basic_adv_contra	85.0	85.4	85.0	87.5	83.8	72.5	5.38	83.20
	**Con-AAE**	**87.5**	**86.7**	**87.9**	**88.3**	**87.1**	**81.7**	2.43	**86.53**
2100	Basic_adv	85.0	85.0	84.2	82.9	84.5	81.4	**1.42**	83.80
	Basic_adv_cyc	72.4	72.4	71.2	70.4	76.2	77.4	2.82	73.33
	Basic_adv_contra	**91.0**	**90.0**	**91.9**	**91.4**	86.2	72.1	7.63	87.10
	**Con-AAE**	89.3	88.8	88.6	90.0	**87.8**	**82.6**	2.67	**87.85**
3000	Basic_adv	81.1	82.5	82.5	81.8	77.2	75.3	3.06	80.07
	Basic_adv_cyc	85.8	86.8	87.0	86.5	**84.0**	80.6	**2.47**	85.12
	Basic_adv_contra	88.8	**90.0**	**89.2**	67.5	60.8	**86.5**	12.87	80.47
	**Con-AAE**	**89.7**	88.6	88.2	**89.1**	74.2	82.0	6.12	**85.30**
6000	Basic_adv	81.0	81.2	74.2	67.5	44.7	35.8	19.33	64.07
	Basic_adv_cyc	81.1	85.2	85.5	78.6	79.2	**78.5**	**3.24**	81.35
	Basic_adv_contra	**90.8**	**90.9**	**91.3**	**90.0**	81.9	67.6	9.43	85.42
	**Con-AAE**	87.5	89.7	88.3	87.3	**82.9**	77.1	4.69	**85.47**

F. Hardware information and running time

We run tools using GPU on a machine cinfigured with one NVIDIA Tesla V100 GPU. For tools using CPU, we implement them in an environment configured with one Intel(R) Xeon(R) gold 6226R CPU @ 2.90GHZ and 240 GB memory. The running time is in table S8. We can see that traditional machine learning tools are faster than deep learning models, even though most of them are implemented on the CPU. Deep learning usually requires a lot of calculations to fit massive parameters, which is a time-consuming process. Benefiting from the development of GPU, the training time of the deep learning model has been significantly shortened,

though it is still slower in most cases. On the other hand, the performance of deep learning methods is usually better than statistical machine learning methods. So there is a trade-off between time and performance. How to design fast and high-performance models is still an important problem.

Table S8. Running time for various methods on different datasets. The unit is seconds.

	Deep learning?	Using GPU?	sci-CAR	SNARE	PBMC	SHARE
Con-AAE	Yes	Yes	4523	1750	6622	10603
Cross-Modal	Yes	Yes	2163	1230	1251	2917
cross-Modal-anchor	Yes	Yes	2287	1260	1292	2869
MMD-MA	No	Yes	161	127	728.6	
SCOT	No	No	5358	3218	119735	
UnionCom	No	No	2025	2100	8803	
Pamona	No	No	6474	1150	4947	
Seurat	No	No	514	1001	3040	
DCCA	Yes	Yes	53	60	300	80
Cycle-Gan	Yes	Yes	3232	164	13480	22531
scJoint	Yes	Yes	78	57	1933	2054
MOFA+	No	No	1973	142	677	1771

G. Con-AAE trained with pairwise information

Table S9. Comparison between Con-AAE and Con-AAE-anchor on sci-CAR dataset. Con-AAE is a very stable unsupervised method, which is robust to noise. So the weak supervision with a high level of noise does not significantly improve Con-AAE's performance.

Method	Integration ACC	Recall@k=10	Recall@k=20	Recall@k=30	Recall@k=40	Recall@k=50
Con-AAE	64.1	5.3	12	17	22.6	28.4
Con-AAE-anchor	63.4	7	13.7	18.4	25.4	29.1

H. Visualization

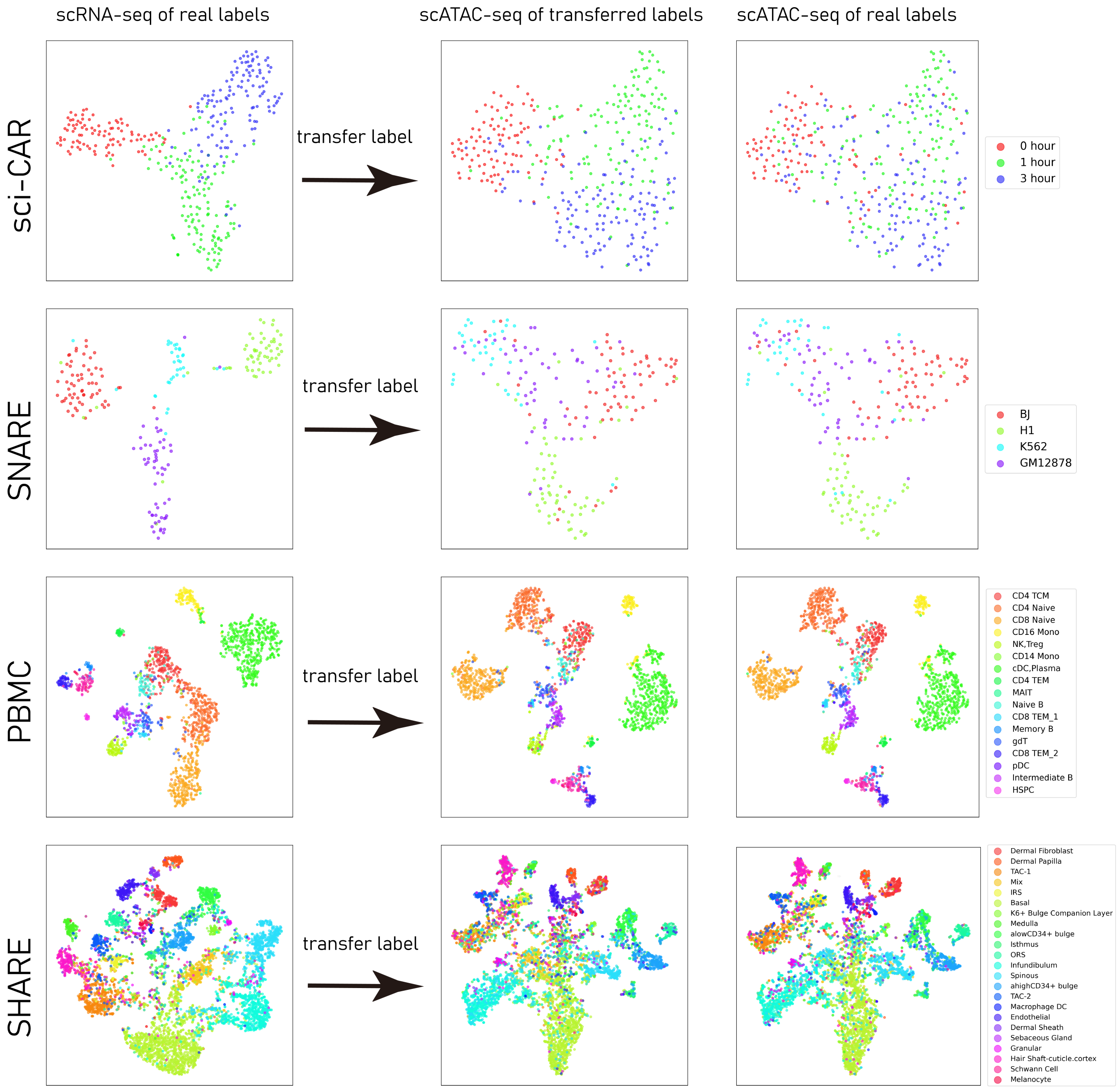

Fig. S1. Label transferring by Con-AAE. The figures in the first column are embeddings encoded from scRNA-seq data of each dataset annotated with real cell type information. The pictures in the second column are embeddings encoded from scATAC-seq data of each dataset annotated with transferred labels. Figures in the third column are ATAC-seq embeddings labelled with real cell type information.

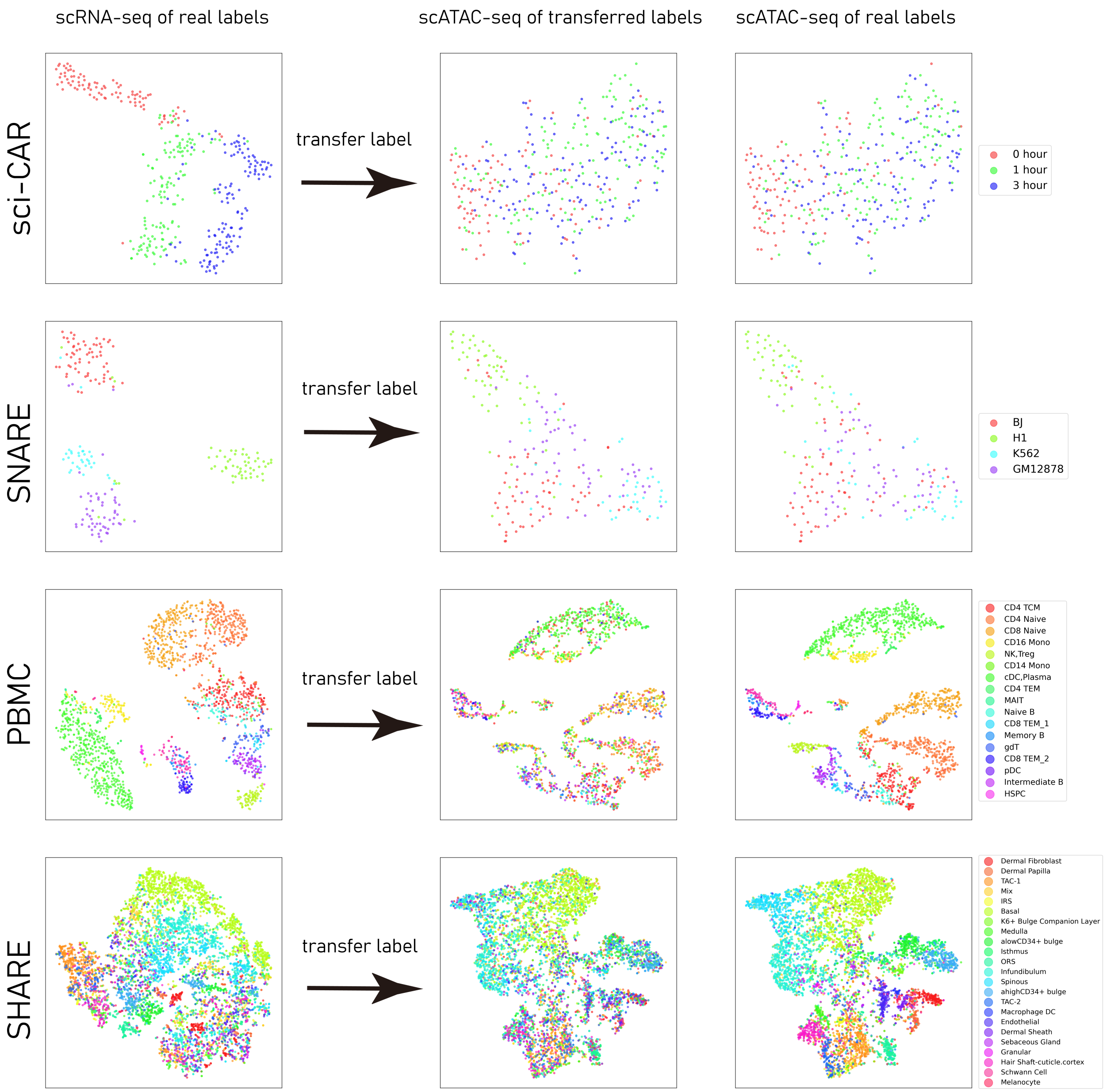

Fig. S2. Label transferring by Cross-Modal.

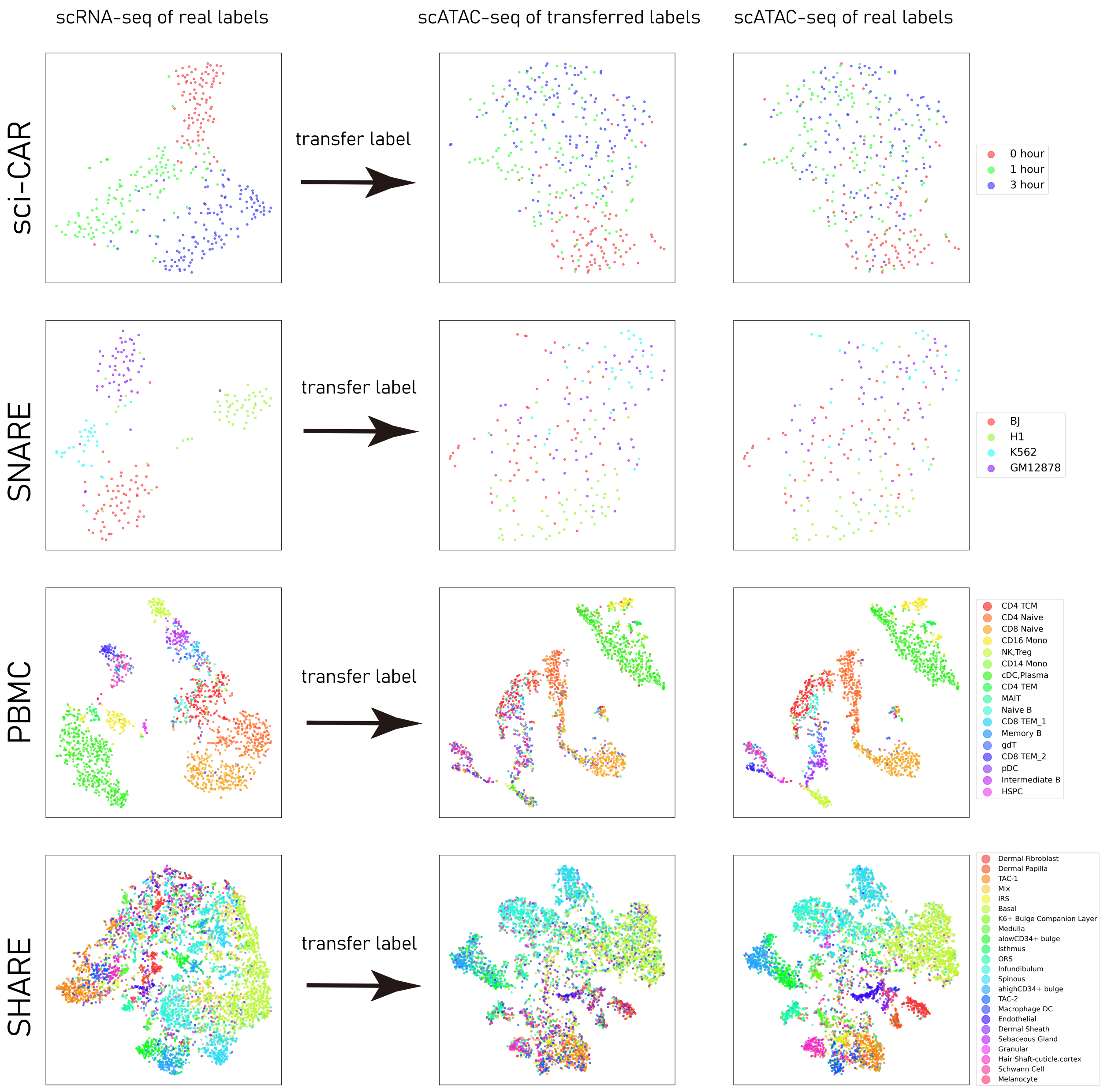

Fig. S3. Label transferring by Cross-Modal-anchor.

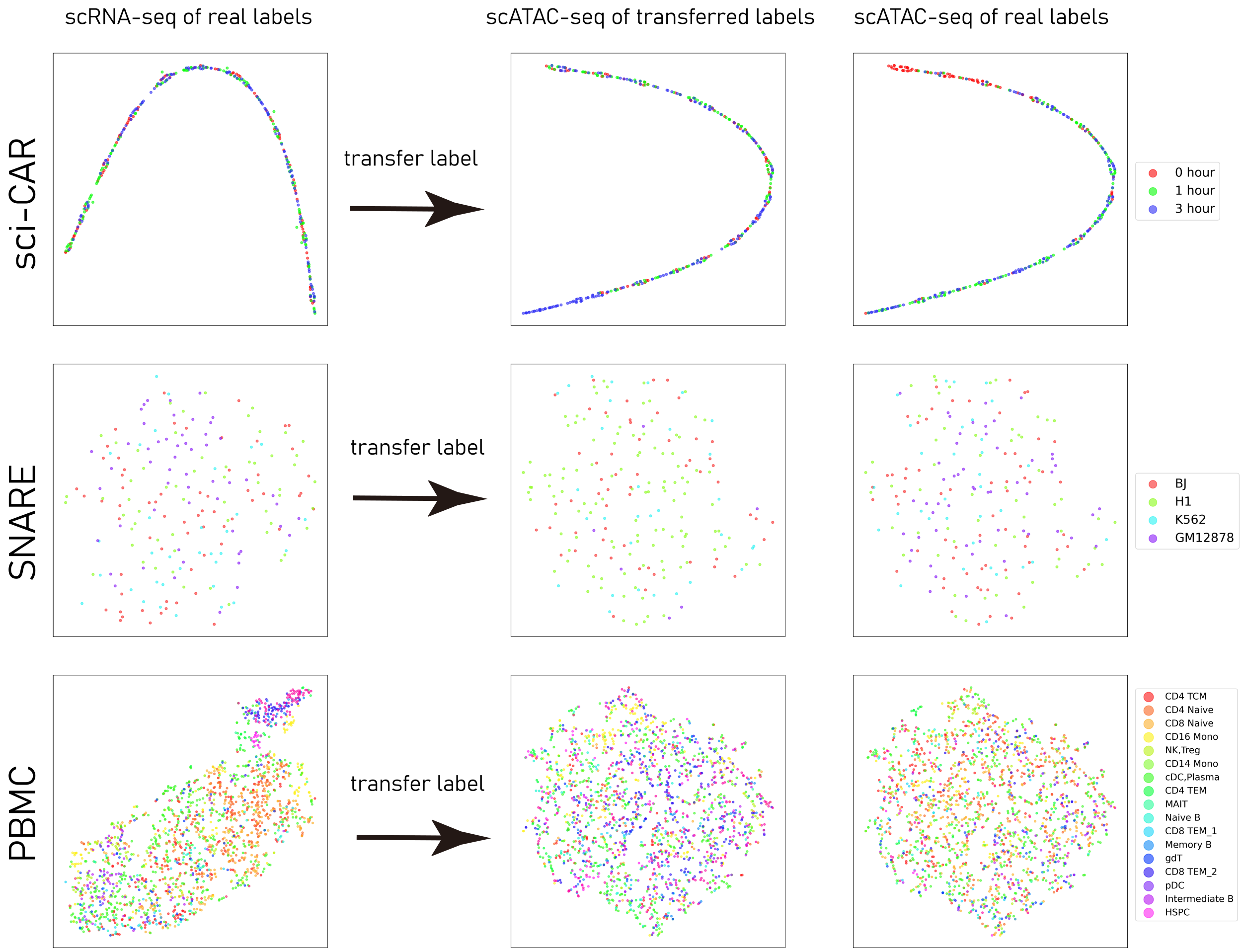

Fig. S4. Label transferring by MMD-MA.

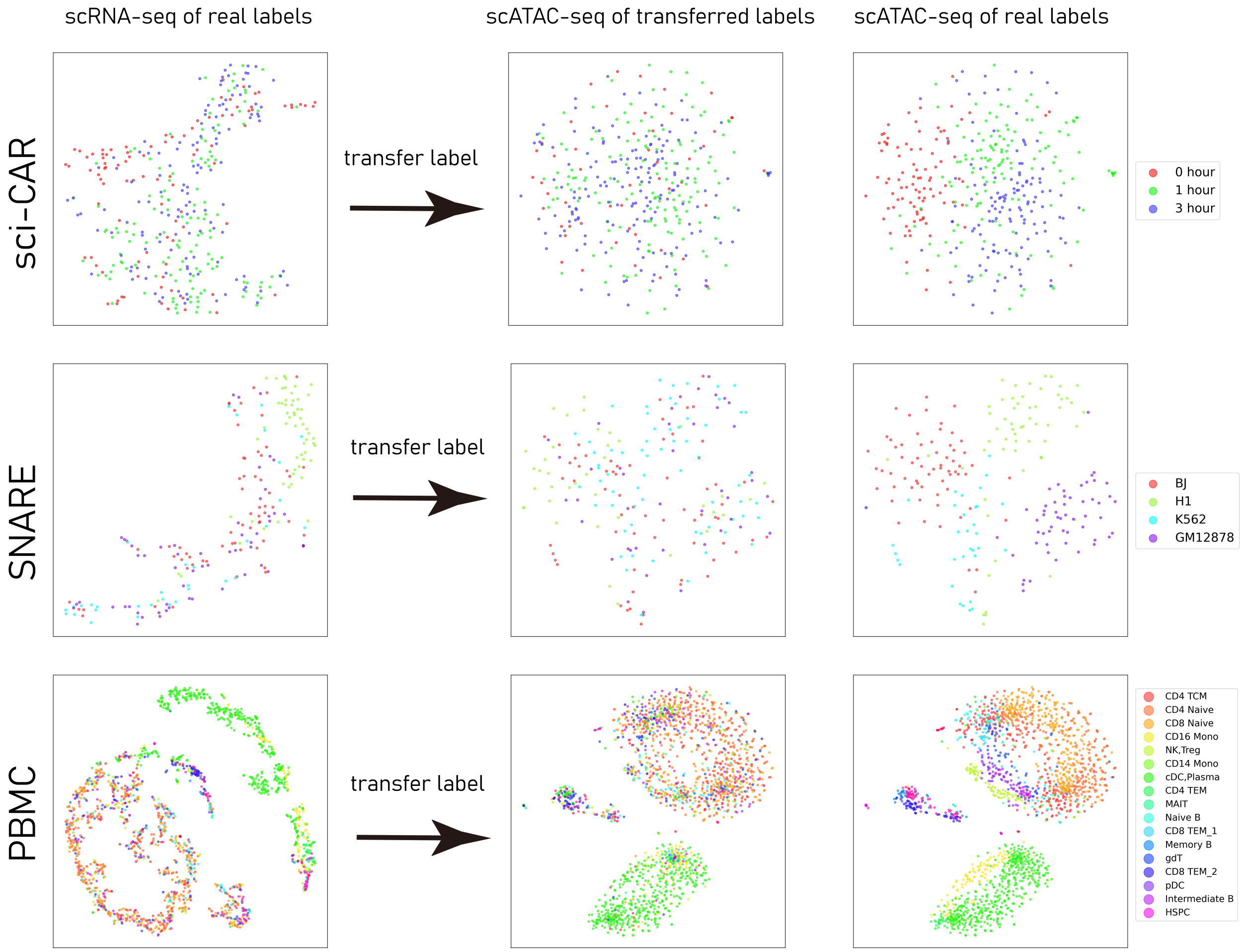

Fig. S5. Label transferring by SCOT.

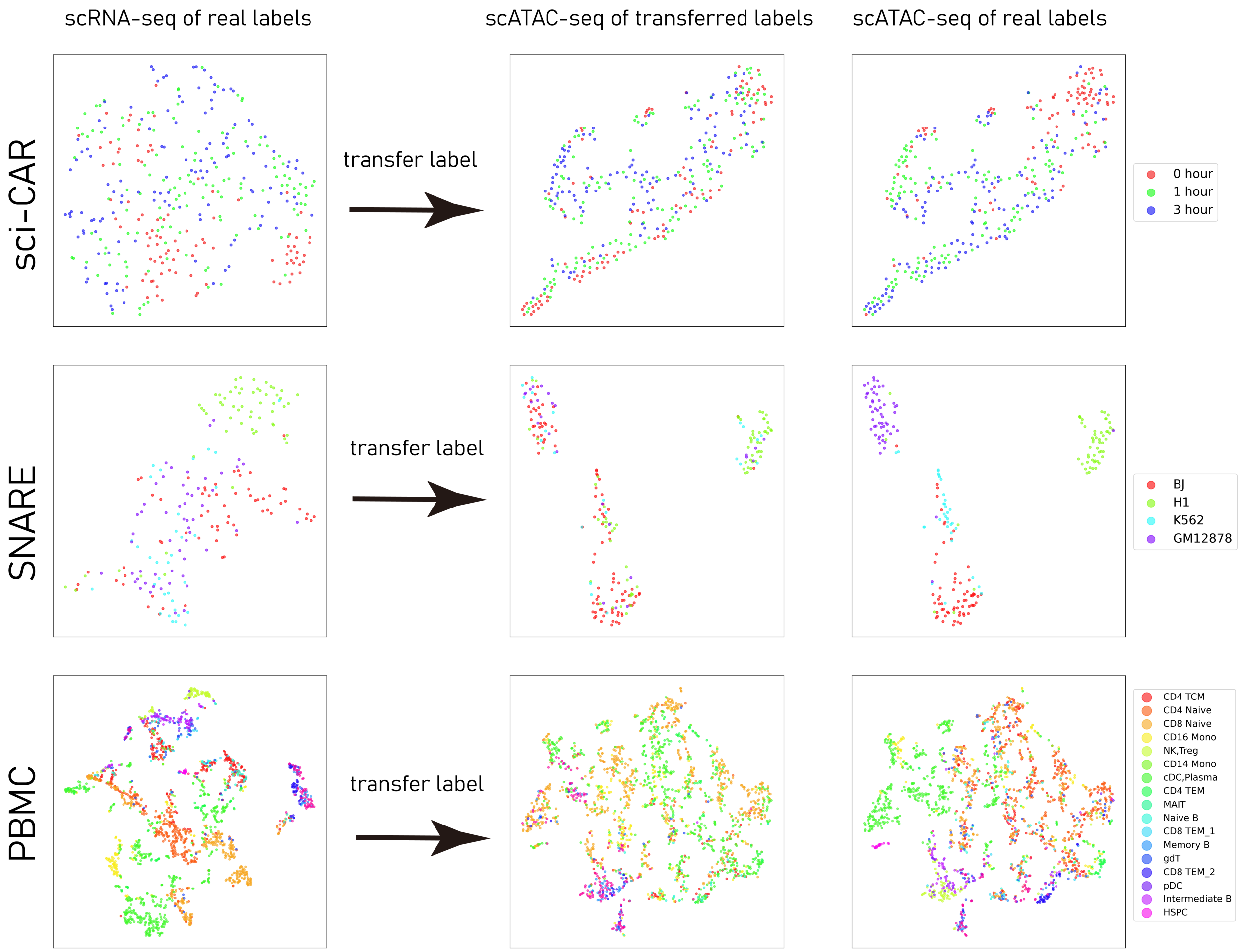

Fig. S6. Label transferring by UnionCom.

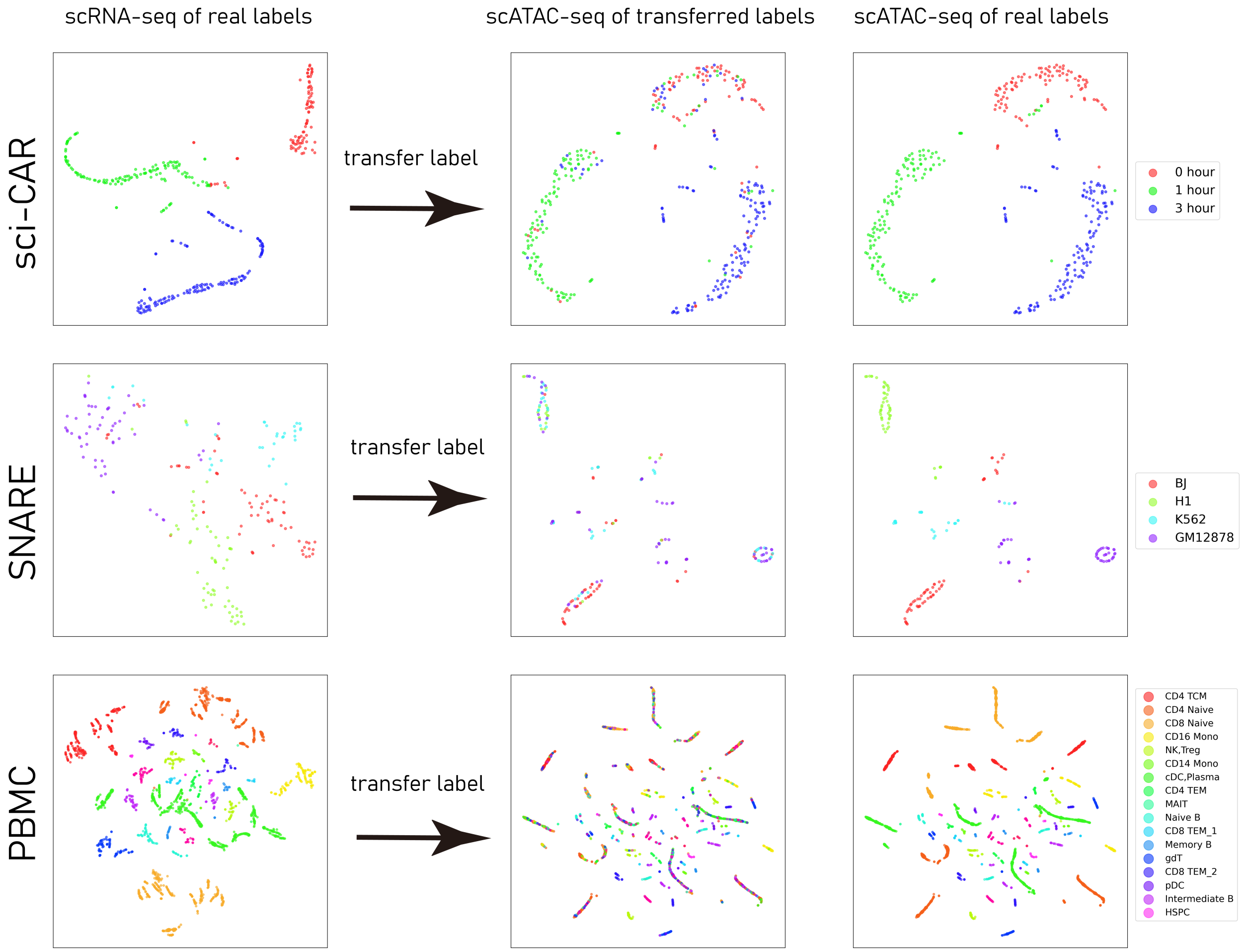

Fig. S7. Label transferring by Pamona.

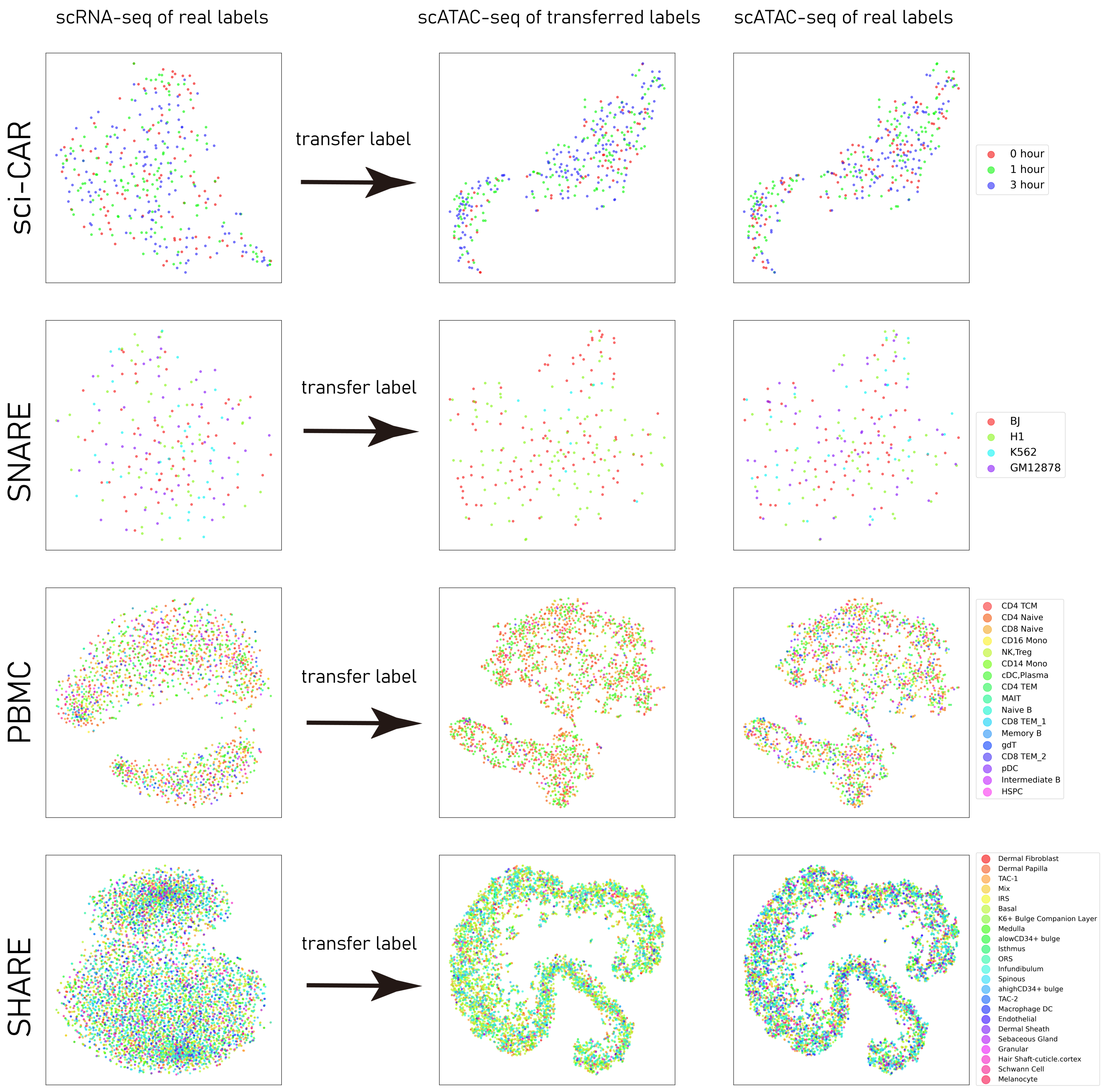

Fig. S8. Label transferring by DCCA.

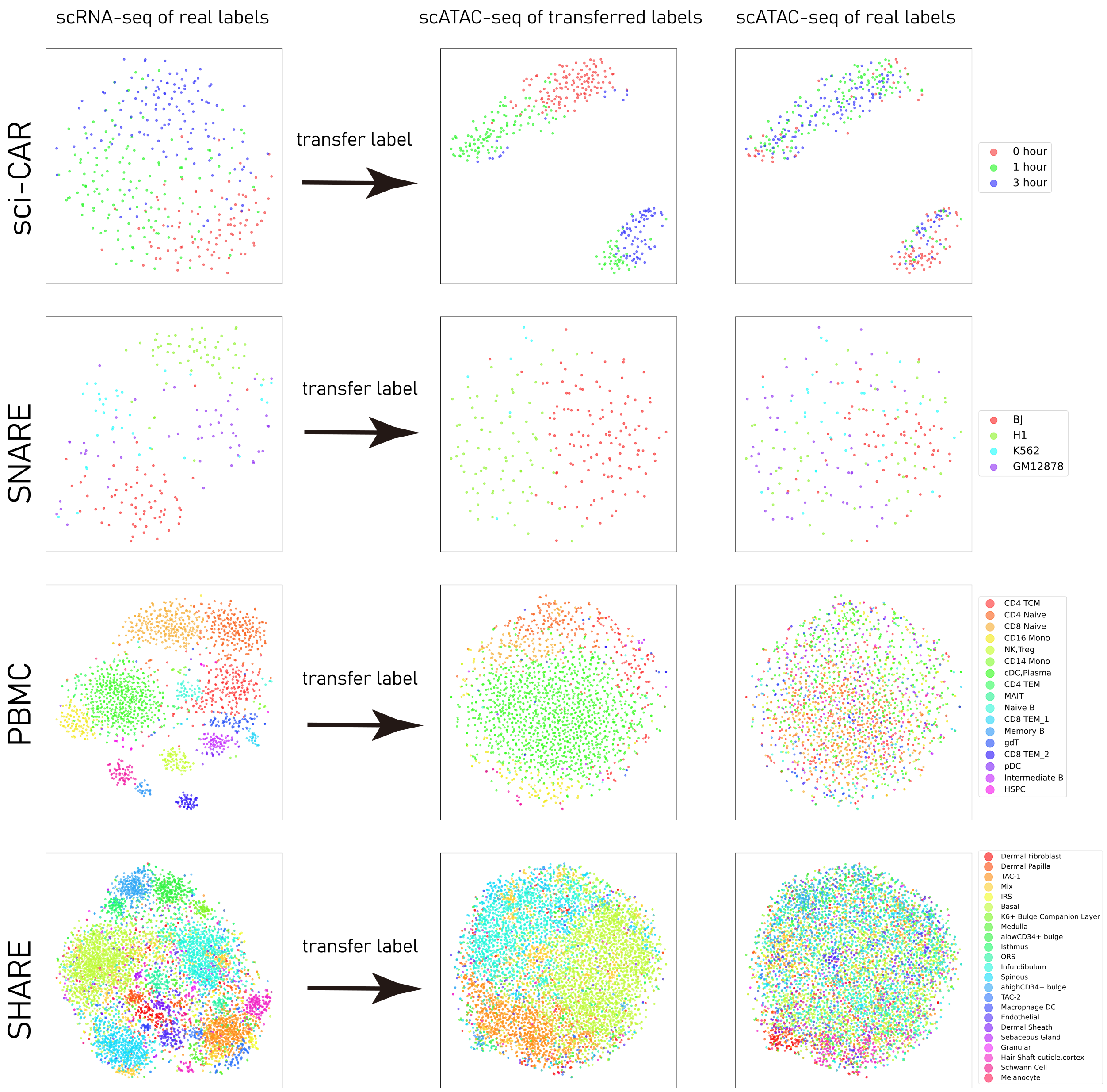

Fig. S9. Label transferring by scJoint.

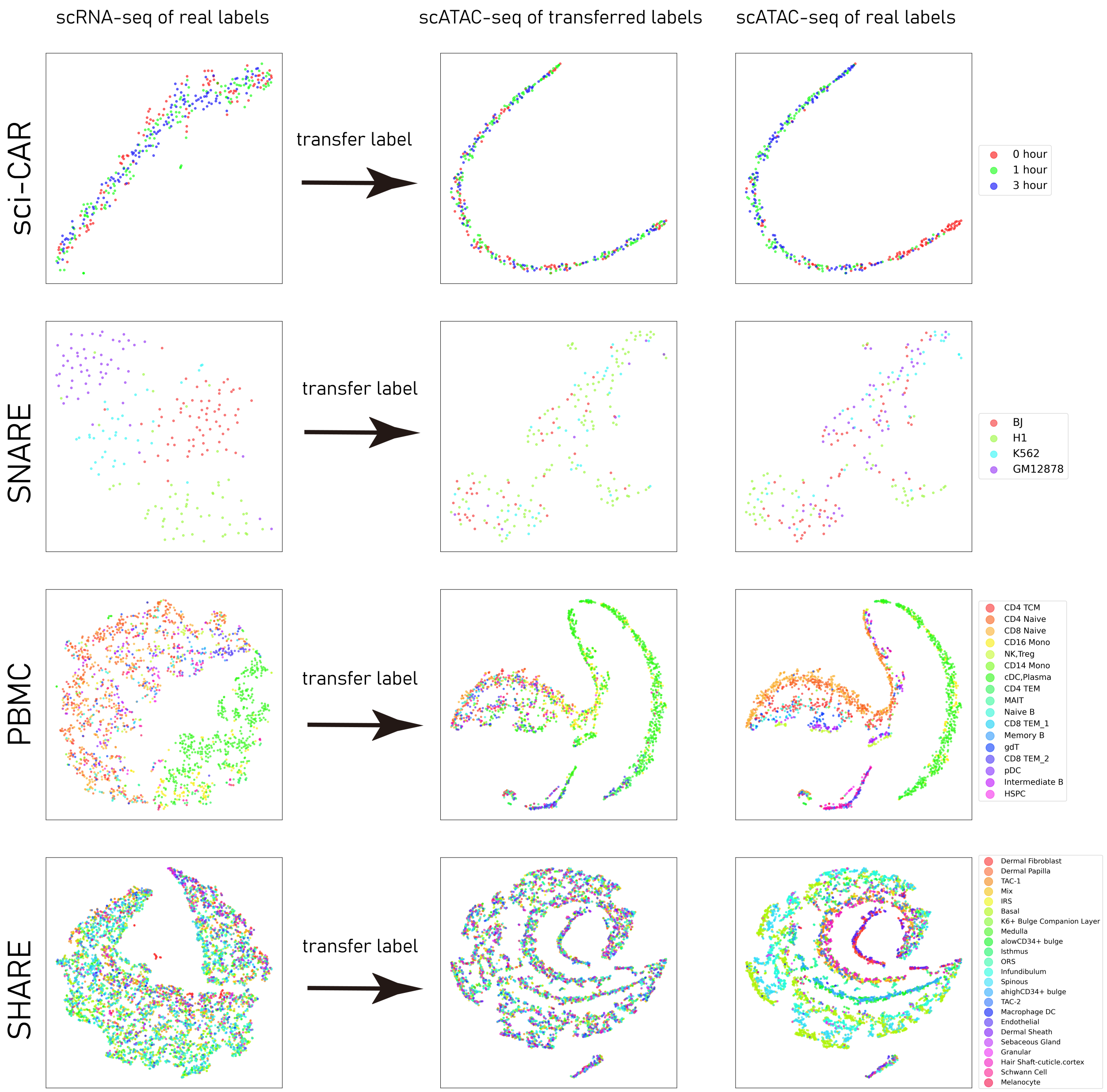

Fig. S10. Label transferring by MOFA+.

REFERENCES

1. J. Cao, D. A. Cusanovich, V. Ramani, D. Aghamirzaie, H. A. Pliner, A. J. Hill, R. M. Daza, J. L. McFaline-Figueroa, J. S. Packer, L. Christiansen *et al.*, “Joint profiling of chromatin accessibility and gene expression in thousands of single cells,” Science **361**, 1380–1385 (2018).
2. K. Dai Yang, A. Belyaeva, S. Venkatachalapathy, K. Damodaran, A. Katcoff, A. Radhakrishnan, G. Shivashankar, and C. Uhler, “Multi-domain translation between single-cell imaging and sequencing data using autoencoders,” Nat. Commun. **12**, 1–10 (2021).
3. S. Chen, B. B. Lake, and K. Zhang, “High-throughput sequencing of the transcriptome and chromatin accessibility in the same cell,” Nat. biotechnology **37**, 1452–1457 (2019).
4. Z.-J. Cao and G. Gao, “Multi-omics single-cell data integration and regulatory inference with graph-linked embedding,” Nat. Biotechnol. pp. 1–9 (2022).
5. S. Ma, B. Zhang, L. M. LaFave, A. S. Earl, Z. Chiang, Y. Hu, J. Ding, A. Brack, V. K. Kartha, T. Tay *et al.*, “Chromatin potential identified by shared single-cell profiling of rna and chromatin,” Cell **183**, 1103–1116 (2020).
6. L. Zappia, B. Phipson, and A. Oshlack, “Splatter: simulation of single-cell rna sequencing data,” Genome biology **18**, 1–15 (2017).
7. J. Herman and W. Usher, “Salib: An open-source python library for sensitivity analysis,” J. Open Source Softw. **2**, 97 (2017).
